# Causes and consequences of tipping points in river delta social–ecological systems

**DOI:** 10.1007/s13280-023-01978-2

**Published:** 2024-04-13

**Authors:** Emilie Cremin, Cai J. T. Ladd, Thorsten Balke, Sumana Banerjee, Ly H. Bui, Tuhin Ghosh, Andy Large, Hue Thi Van Le, Kien V. Nguyen, Lan X. Nguyen, Tanh T. N. Nguyen, Vinh Nguyen, Indrajit Pal, Sylvia Szabo, Ha Tran, Zita Sebesvari, Shah Alam Khan, Fabrice G. Renaud

**Affiliations:** 1 School of Social and Environmental Studies, The University of Glasgow, Dumfries Campus, Rutherford/McCowan Building, Crichton University Campus, Dumfries, DG1 4ZL Scotland, UK; 2 School of Geography and Earth Science, University of Glasgow, Glasgow, UK; 3 University of Swansea, Swansea, UK; 4 School of Oceanographic Studies, Jadavpur University, Kolkata, India; 5 VNU-Central Institute for Natural Resources and Environmental Studies (VNU-CRES), Vietnam National University (VNU), Hanoi, Vietnam; 6 School of Geography, Politics and Sociology, Newcastle University, Newcastle upon Tyne, UK; 7 Research Center for Rural Development, An Giang University, An Giang, Vietnam; 8 Disaster Preparedness, Mitigation and Management, Asian Institute of Technology, Pathum Thani, Thailand; 9 Australian National University, Canberra, Australia; 10 United Nations University, Institute for Environment and Human Security, Bonn, Germany; 11 Bangladesh University of Engineering and Technology, Dhaka, Bangladesh; 12 Department of Development and Sustainability, Dongguk University, Seoul, South Korea; 13 Ostrom Center for the Advanced Study in Natural-Resource-Governance, Pathum Thani, Thailand; 14 Can Tho University, Can Tho, Vietnam

**Keywords:** Cascading effects, River deltas, Social–ecological systems, Sustainable development, Systematic literature review, Tipping points

## Abstract

The sustainability of social–ecological systems within river deltas globally is in question as rapid development and environmental change trigger “negative” or “positive” tipping points depending on actors’ perspectives, e.g. regime shift from abundant sediment deposition to sediment shortage, agricultural sustainability to agricultural collapse or shift from rural to urban land use. Using a systematic review of the literature, we show how cascading effects across anthropogenic, ecological, and geophysical processes have triggered numerous tipping points in the governance, hydrological, and land-use management of the world’s river deltas. Crossing tipping points had both positive and negative effects that generally enhanced economic development to the detriment of the environment. Assessment of deltas that featured prominently in the review revealed how outcomes of tipping points can inform the long-term trajectory of deltas towards sustainability or collapse. Management of key drivers at the delta scale can trigger positive tipping points to place social–ecological systems on a pathway towards sustainable development.

**Supplementary Information:**

The online version contains supplementary material available at 10.1007/s13280-023-01978-2.

## Introduction

Anthropogenic impacts on the environment are reaching a point where the Earth’s biosphere systems are passing dangerous tipping points (Lenton et al. 2008; Rockström et al. 2009; Willcock et al. 2023). Once these points are crossed, the climate that has supported a stable environment throughout human existence begins to break down (Steffen et al. 2015a, 2015b). There is evidence that several global climate-related tipping points, including the collapse of glaciers, melting of carbon-rich permafrost, and coral die-off, have already been crossed (Armstrong McKay et al. 2022; Kemp et al. 2022). But climate change is just one of the many drivers of risk. Many new risks emerge when and where our physical and natural worlds interconnect with human society (Eberle et al. 2023). The risk of reaching tipping points becomes more likely when compounding and interconnected drivers of tipping points such as groundwater depletion, saline water intrusion, decline in sediment deposit, land conversion, and increased pollution in river deltas, are considered (Hillebrand et al. 2020; O’Connor et al. 2021; Eberle et al. 2023; Willcock et al. 2023). The likely consequence of crossing these tipping points are coastal erosion, loss of land and livelihoods, water and food insecurity and ultimately mass displacement of people (Berchin et al. 2017; McLeman 2018; Adams and Kay 2019; Lincke and Hinkel 2021) that will undermine global efforts towards achieving the UN Sustainable Development Goals (SDGs) for bringing about positive outcomes for humankind (Szabo et al. 2016a).

Complex social–ecological systems (SESs) of the world's mega-deltas (i.e. deltas with areas above 1000 km^2^ according to Syvitski et al. 2022), are at the frontline of climate and environmental change and could be particularly prone to tipping points and their negative consequences. Approximately 339 million people now live in deltas (McCracken and Wolf 2019; Edmonds et al. 2020) and these landforms produce a significant proportion of the world’s food (Renaud et al. 2013). The rapid rise in human population and associated demand for resource extraction combined with major biodiversity loss has dramatically altered delta environments, triggering environmental degradation (Kuenzer and Renaud 2012; Moder et al. 2012; Best and Darby 2020), loss of livelihoods (Hill et al. 2020; Kuenzer et al. 2020), and *in extremis* could lead to the potential collapse of intertwined delta SESs (Renaud et al. 2013; Best 2019; Edmonds et al. 2020). Wang et al. (2012) argue that there is a need to anticipate tipping points, or critical transitions, in social–ecological systems; it is imperative to understand better how anthropogenic and naturogenic activities instigate large-scale change in river deltas, and whether these changes threaten the crossing of irreversible tipping points (Norgaard et al. 2021).

In the context of SESs, tipping points can be defined as a breakpoint between two system states which can be reached when major and controlling variables of an SES no longer support the prevailing system and the entire system shifts to a new, distinct state defined-by a new set of boundary conditions (Renaud et al. 2013; Milkoreit et al. 2018). In river deltas, tipping points have been described as operating over vastly different scales of space and time—from the rise and fall of sea levels over geological timescales driving transitions in coastal delta habitats (Lenton et al. 2008; Törnqvist et al. 2020) to the scale of individual’s decisions on whether to stay or migrate in the face of disturbance (Milkoreit et al. 2018; Winkelmann et al. 2022). One widely reported current tipping point in Asian deltas is the widespread conversion of rice paddies to shrimp ponds over the last two decades. Whilst the conversion of rice paddy to shrimp pond is comparatively simple in land-use terms, reversion to rice paddy is made extremely difficult by subsequent salinisation of water and soil to the point where rice cannot grow. Once the new ‘aquaculture’ state is established, the controlling variables that govern the new state (i.e. salinisation) are largely irreversible (Dang 2020; Kruse et al. 2020). However, an actor’s position and perception of whether a tipping point is positive, or negative is subjective, nuanced, and may change over time. Transitions to aquaculture, for example, have resulted in the short-term positive economic boom that shrimp production provides (Lebel et al. 2002) versus long-term negative decline in rice production and availability of freshwater due to salinisation of soil and water (Renaud et al. 2015). Rising population densities, increased urbanisation or crop production have led to a capital gain in many deltas (Garschagen et al. 2011; Loucks 2019; Kuenzer et al. 2020).

Since the development of tipping point theory and the demonstration of their existence in social–ecological systems (Holling 1973; Garschagen 2010; Lenton 2011; Nuttall 2012), there has been a rapid increase in research efforts to document tipping points in the world’s river deltas (Milkoreit et al. 2018; Lauerburg et al. 2020). Here, we present the first systematic review of the literature to identify the causes and consequences of delta-specific tipping points, thereby addressing a significant knowledge gap for these highly important yet highly vulnerable environments. By understanding *why* tipping points happen—and potential trajectories towards them i.e. *how*—steps can be taken to help promote transformations that benefit delta ecosystems and societies and avoid ones that harm them (Moore et al. 2014; van Ginkel et al. 2020; Winkelmann et al. 2022). The aims of this global review are to identify the causes, consequences, and nature of tipping points occurring within river delta SESs and identify key recommendations to both avoid the negative tipping points and enhance positive ones.

## Methods

### Literature review

This paper comprises part of the research programme of the UKRI GCRF Living Deltas Hub (2019–2024) project and was specifically designed as a unique knowledge co-production exercise involving researchers on an equitable (gender, regional appartenance and career stage) basis. Web of Science and SCOPUS databases were used to identify peer-reviewed literature on social–ecological tipping points in river deltas published from 01/01/1989 to 19/10/21. The review includes all study types (including reviews, numerical models, and commentaries). All papers chosen for inclusion had to explicitly examine tipping points in social, ecological, or coupled social–ecological systems of the world’s river deltas. Given that multiple terms exist to describe tipping points across disciplines, we used keywords identified by Milkoreit et al. (2018) in our keyword search (Appendix SM1.a.). We used three levels of screening (title, abstract, and full text) to identify appropriate papers for this review. At each level, papers were excluded if they did not consider social–ecological tipping points in river deltas, either directly or indirectly, and were in a language other than English. We recorded the number of studies included or excluded in the analysis, and the reasons for exclusions according to the Preferred Reporting Items for Systematic Reviews and Meta-Analyses (PRISMA) Statement (Moher et al. 2016) (Appendix SM1.b.). Of the 4560 papers identified in the initial keyword search, 77 were selected for qualitative synthesis.

### Data extraction

From the articles selected for qualitative synthesis, we extracted the year of publication, delta location, the temporal and spatial domain of the study, the tipping point described, the lead-up root causes of the tipping point, the consequences of the tipping point, whether those consequences were described as negative or positive, and any recommendations made on avoiding negative or promoting positive tipping points. A unique record was created when articles considered more than one tipping point or delta.

Each record was assigned a unique spatial (“Patch”, “Local”, “Sub-Delta”, “Delta”, or “Basin”) and temporal (“Seasons”, “Years”, “Decades”, “Centuries”, or “Millennia”) domain. Keywords relating to the cause, tipping point, and consequence were standardised. For example, “salinisation” was used as the standard term, which included “salinity intrusion”, “saline intrusion”, and “salinization”. Keywords were chosen after reading the papers and allocating delta-relevant information into categories, according to common themes within the causes (“Social”, “Ecological”, “Environmental”), tipping points (“Systemic”, “Governance”, “Hydrological”, “Food production”, “Flood management”), and consequences (“Positive”, “Negative”, “Both”) categories. Flows between causes, tipping points, and consequences were also noted per record, enabling the often-hierarchical connections between keywords to be delineated. For example, a hierarchical chain may be “climate change → sea level rise → salinisation [causes], agriculture → aquaculture [tipping point] → economic growth [consequence]”. Counts of the number of times a keyword featured in a record (nodes), and the number of times a unique connection existed between nodes (links) were recorded.

Each paper was reviewed by at least two GCRF Hub co-authors from different regions, gender and career stage, and records were considered finalised once both co-authors agreed on the entries. To standardise data entries, the two lead authors performed a quality check on all reviewed papers to assign causes, tipping points, and consequences to each category. Papers used in the review are cited by a unique identifier number (see column “ID” of the reference list in Appendix SM2).

## Results

### General overview

**Fig. 1:**
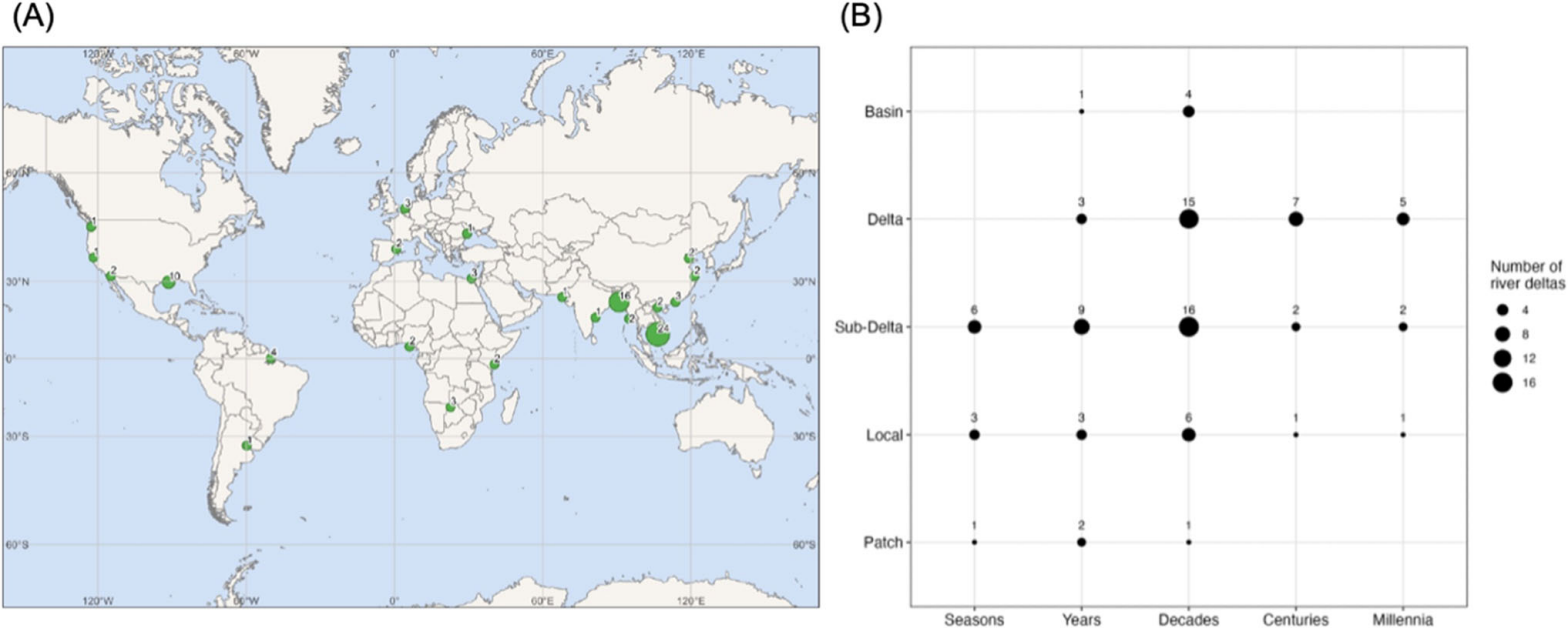
Locations of river deltas where tipping points have been described (**A**). Location of the river deltas and number of papers select for each delta over which tipping points were studied (**B**). The numbers and size of points relate to the number of times a delta is featured in the review from the scale of a patch (ecosystem) to the scale of a river basin, and from seasons to millennia

The number of studies examining tipping points in river delta social–ecological systems has grown rapidly since 2005 (Appendix SM1.c.). The earliest paper contained in the literature review was Admiraal et al. (1989) after the formal concept of tipping point theory was described by Holling (1973). The subsequent rise in publications, starting from the mid-2000s, likely coincided with the seminal work of Scheffer and Carpenter (2003) on detecting tipping points. Deltas featured in this review had global coverage. Twenty-two deltas were analysed, and the Mekong River Delta (MRD) (*n* = 24), Ganges–Meghna–Brahmaputra (GBM) (*n* = 16), and Mississippi–Atchafalaya–Wax (MAW) (*n* = 10) mega-deltas had the highest representation in the literature (Fig. 1a). Studies considered tipping points across a range of spatial and temporal scales, with most studies describing tipping points at decadal and sub-delta to delta scales (Fig. 1b).

### Characterising tipping points 

**Fig. 2:**
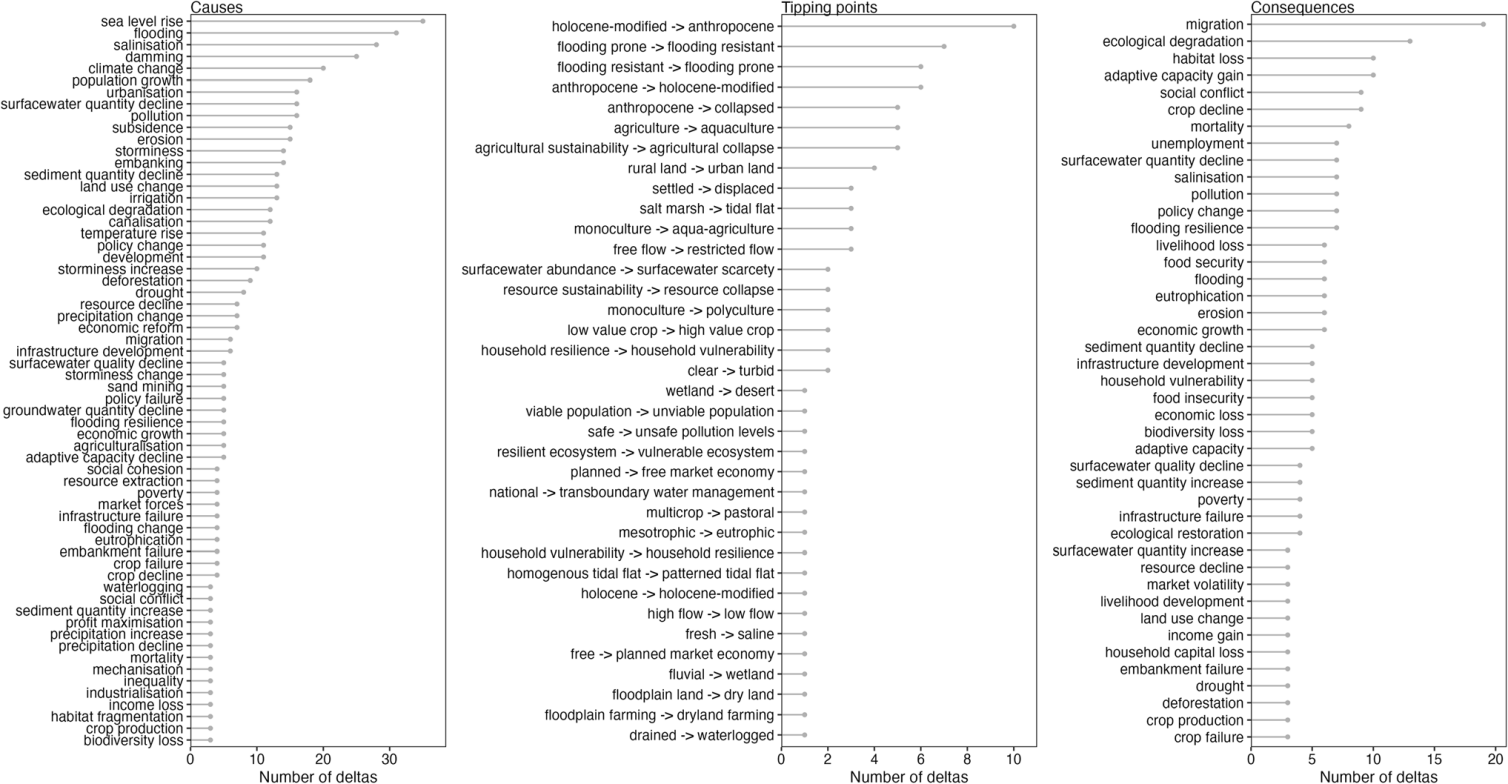
Frequency of cause, tipping point, and consequence keywords cited in this review. Only keywords that were mentioned three times or more for cause and consequence are shown. See Appendix SM2 for a complete list

A total of 36 major tipping points were identified (Fig. 2). Tipping points frequently described systemic shifts (e.g. Holocene-modified to Anthropocene delta state), changes in flood management (e.g. flooding prone to flooding resistant), and land-use change (e.g. agricultural sustainability to agriculture collapse).

Systemic shifts represented the cumulative effect of human and ecological modifications to SES processes operating at larger (sub-delta to basin) scales. The transformation from a ‘Holocene-modified’ to an ‘Anthropocene’ state, for example, was characterised by a net human control driving SES processes, such as the reduction in sediment delivery leading to increased land subsidence and rapid coastal erosion (see references of paper IDs 22, 43, 44, 46, 49, 57, 58, 60, 61, 66 in Appendix SM2). Transformations from ‘Anthropocene’ to ‘Holocene-modified’ states in the other direction were also reported, such as through the restoration of wetlands and rivers (53, 35, 31, 44, 38, 8). Transitions in flooding risk and management represented ‘flooding resistant’ transformations, represented by measures taken to protect against flooding such as the construction of defences (4, 7, 26, 32, 40, 51, 59). Shifts to ‘flooding prone’ were represented by cases when embankments were breached, changes to river and intertidal geomorphology which caused flooding (19, 20, 33, 41, 65, 76), or where rivers became restricted (62, 68, 69) and saltmarshes transitioned to tidal flats (21, 37, 55) leading to increased flood risk. Several studies also reported major land-use transitions such as regime shifts from agricultural sustainability to agricultural collapse (23, 30, 34, 56, 67), agriculture to aquaculture (2, 3, 47, 48, 77), rural to urban land (24, 71, 87, 88), monoculture to aquaculture (82, 17, 18), or monoculture to polyculture (13, 39). Another important transition, frequently described, included a shift from settled livelihoods to displacement or migration (72, 79).

### Causes of tipping points

**Fig. 3:**
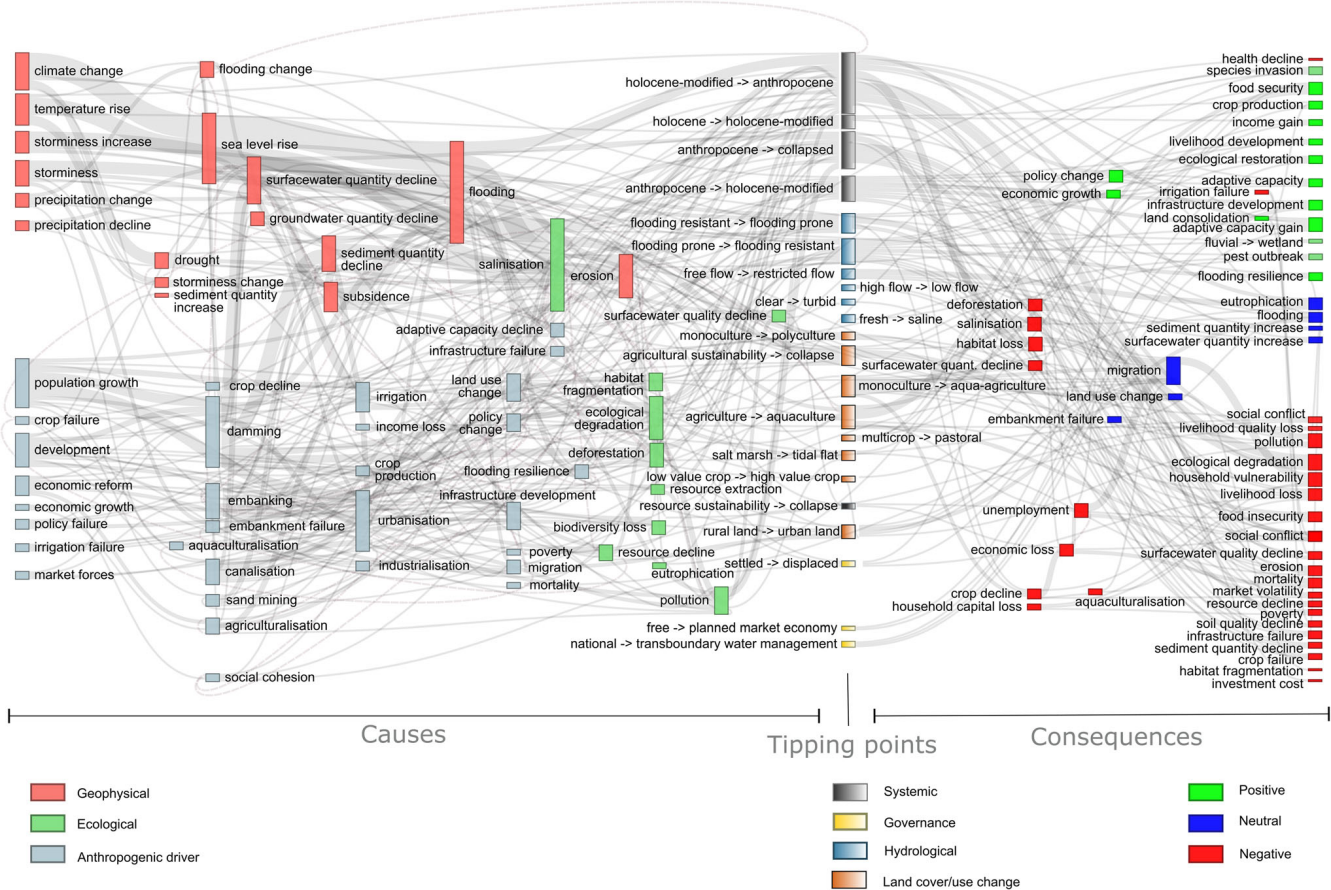
Relationships between the causes and consequences of tipping points for all deltas featured in this review. Node size is proportional to the number of times the keyword was cited per record. Dashed lines represent feedback loops

A total of 62 tipping point drivers were identified (Fig. 2: keywords that appeared three times or more). Tipping points were often caused by a cascade of drivers and were grouped into geophysical, ecological, and anthropogenic classes (Fig. 3).

The three main tipping point drivers were sea level rise, flooding, and salinisation. Climate change impact was identified as the most common root cause of many tipping points, with temperature rise (25, 34, 46, 50), precipitation change (24, 26, 53), increase in storminess (24, 19, 20, 44, 53, 72, 74, 75), sea level rise (2, 3, 9, 13, 23, 24, 26, 34, 35, 39, 19, 20, 44, 46, 50, 53, 64, 72, 77, 82), coastal and river flooding (3, 7, 19, 20, 23, 26, 32, 34, 35, 38, 40, 42, 43, 44, 53, 60, 61, 66, 72), and drought (13, 26, 44) being frequently reported. Geophysical and climate drivers were reported to cause cascading effects that eventually triggered a tipping point in the delta in question. For example, temperature rise leading to drought (26, 44, 46), and drought leading to a decline in crop yield (86), agricultural collapse (34), or soil salinisation (3, 61).

Proximate causes of tipping points were frequently related to changes in water availability (2, 3, 9, 12, 17, 28, 52, 73) and sediment flux (3, 8, 10, 11, 17, 26, 49, 50, 60, 85). Whilst soil erosion in the river basins has provided the sediments to the deltas (McNeill et al. 2017; Lenard et al. 2020), sediment eroded due to human actions on the slopes in recent times (Nienhuis et al. 2020) is compensated by sediment retention due to dams (Darby et al. 2015; Dunn et al. 2019). Consequently, many river deltas across the globe are sediment-starved and have seen a reduction in their size (Hoitink et al. 2020; Scown et al. 2023; Zăinescu et al. 2023).

Urbanisation, infrastructure development, and economic growth were frequently represented as anthropogenic causes of delta SES tipping points (Figs. 2, 3). Economic opportunities driven by market forces (78), economic reform (39, 82, 83), and management and policy successes and failures (18, 29, 48, 59, 64, 70, 77, 81, 86) were recorded as leading to rapid urbanisation in deltas (50, 64, 77). Land-use transformations were often caused by changes in flood management practice (46, 58, 60, 61, 66). Urbanisation is frequently linked to hydraulic development (dams, embankments, dikes, and canals for irrigation or drainage). These infrastructures allow industrialisation (12, 28, 70) which involves migrations (9, 87, 88). Development of deltas has resulted in land-use change (22, 39, 43, 49, 57), intensification of agriculture (70, 82), and extension of cultivated areas over previously non-cultivated lands (agriculturalisation) (6, 39, 82) directed at resource extraction (50), economic growth (56) and population growth. These activities have culminated in the retention, diversion and disruption of water and sediment flux (3, 17, 18, 26, 49, 50, 53, 60, 82), degradation of ecosystems (32, 38, 77), erosion (11, 21, 31, 38, 49, 53, 60, 64), and salinisation (2, 3, 8, 9, 10, 13, 16, 17, 18, 24, 39, 74, 77), acidification (39,50), pollution (50, 57) and subsidence (10, 11, 21, 26, 49, 82) of the delta, triggering tipping points.

Construction of new infrastructure and failure of existing ones (4, 8, 24, 26, 59) both resulted in tipping points. Changes in market forces, sometimes because of economic development, led to price decline (86). The migration of agricultural workers to urban areas, and subsequent urbanisation of the delta, supported the development of the economically attractive aquaculture sector (9). The intensification of aquaculture depended on infrastructure development (39) including the construction and maintenance of canals initially installed to improve water management for irrigation, whilst elsewhere embankments were a common engineering solution to ensure the protection of cultivated areas (3, 9, 11, 17, 18, 22, 39, 43, 68, 69) and settlements (11, 19, 20) from natural hazards.

### Consequences of crossing tipping points

Our review highlighted 43 consequences following tipping points (Fig. 2; keywords that appeared three times or more). Consequences were also observed as cascading, with positive, negative, or neutral outcomes as defined by the authors featured in this review (Fig. 3). The most frequently cited consequences of tipping points were migration and ecological degradation, followed closely by habitat loss, adaptive capacity gain, social conflict, and crop decline. The general trend in the papers reviewed was of positive economic (economic growth, income gain) and social (adaptive capacity gain, food security, flooding resilience) gain versus negative ecological (deforestation, habitat loss, ecological degradation) and environmental quality decline (salinisation, surface water quantity decline, pollution) decline. This pattern was not systematically observed, however, as consequences of tipping points were often connected (e.g. economic growth vs. economic loss; crop production vs crop decline; food security vs food insecurity).

### Regime shifts and feedback loops

**Fig. 4:**
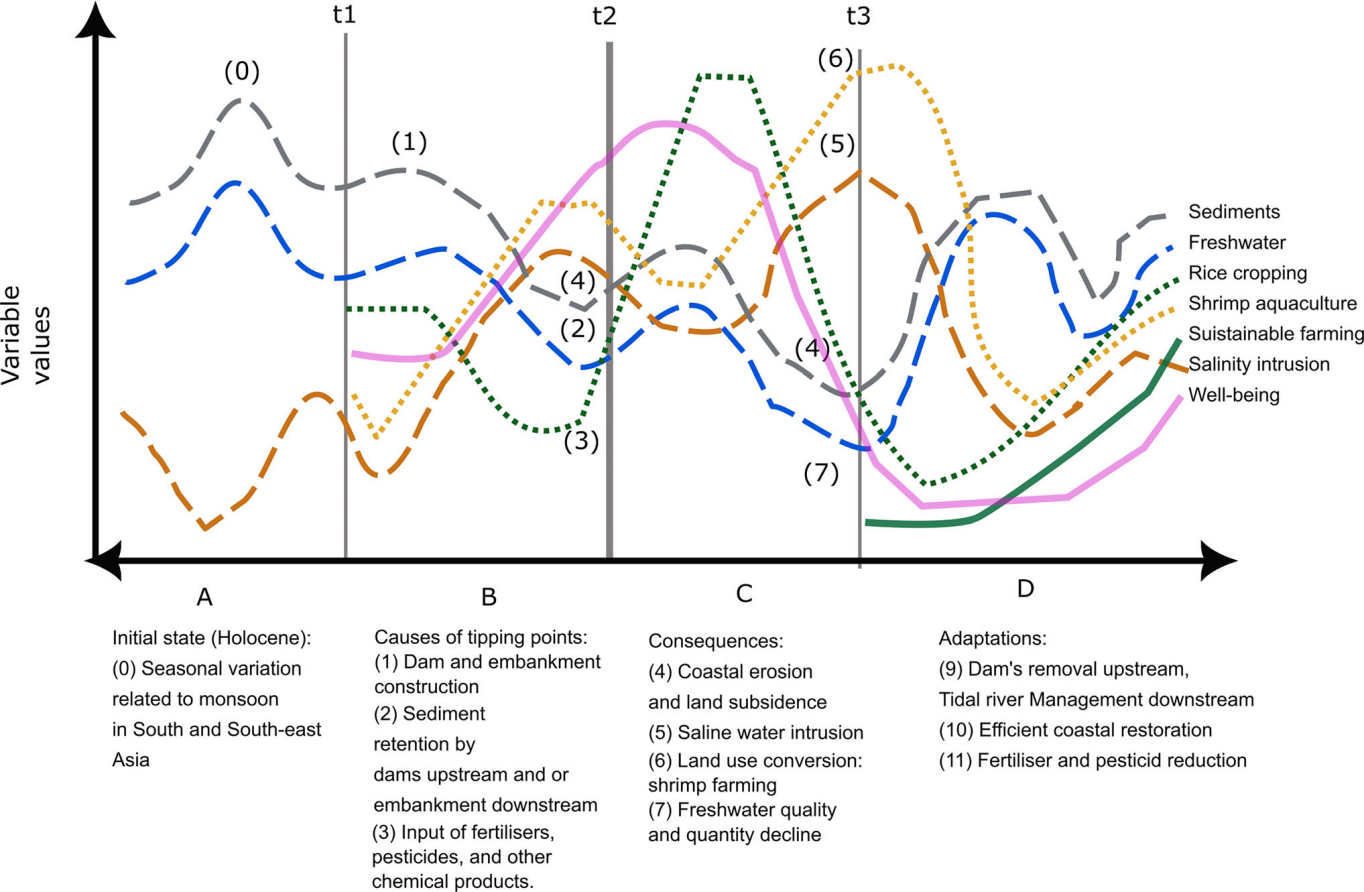
Feedback loops and regime shifts in River delta's Social-ecological systems. Examples from South and South-east asian river basins coastal areas: t1 start of anthropogenic interventions on the earth system in the Holocene. t2 When multiple processes combine and result in compounded tipping points. t3 Start of the adaptation process and resilient transformation. (A) Prior to t1, water flow, sediment discharge and salinity intrusion are related to the seasonality. The salinity intrusion rises during the dry season and declines during the monsoon (0). (B) Between t1 and t2, the seasonality still occurs, but it is modified by the construction of dams and embankments (1). The system began degrading due to increased unsustainable human interventions. The consequence leads to tipping points: t2. (C) After crossing the t2 threshold, we observe a regime shift with feedback loops: Sediments retained by infrastructures (2) lead to coastal erosion (4). Water retained by infrastructure (2) leads to increased salinity intrusion (5). Input of fertilisers, pesticides, and other chemical products (3) leads to water quality decline (7). Moreover, salinity intrusion is enhanced by the increase exacerbated by the land-use conversion from rice cropping to shrimp farming. Social well-being may improve at the outset, but decline with the decline of ecological functionalities and the loss of ecosystem services. (D) If a social reaction takes place at t3 then the implementation of integrated sustainable environmental management, including dam removal (9), efficient coastal restoration (10), and reduction of fertilisers (11) can potentially lead to adaptation and resilience

The key feedback loops comprise processes leading to, and resulting from, tipping points and consequent regime shifts as these either stabilise or destabilise the state of an SES. A range of ecosystem-scale feedback loops were found in the literature and social systems and ecosystems were seen to be exposed to different levels of environmental or economic stress, including multiple components (see SM2, Fig. 4). For example, soil salinisation leads to land-use change (46, 47, 49, 61, 72) and land-use change results in further salinisation. Feedback loops were identified in both ecological (e.g. climate, water, salinity) and social (e.g. farming production and subsidy) components of river delta SESs.

In Fig. 4 we present the trends of a selection of variables (sediments, freshwater, rice cropping, shrimp aquaculture and salinity intrusion) identified in the literature review as major indicators sensitive to anthropogenic actions and involved in regime shifts (4, 77). Figure 4 starts with a seasonal cycle involving the variation in freshwater and sediments carried by the river discharge (Fig. 4A). Figure 4 then illustrates how human intervention started to modify the initial “delta dynamic metastable equilibrium” and to divert the cycles from their seasonal trajectories (Fig. 4). When dams appear on the river network, river flow and sediments are retained by the dams, especially during the dry season. Consequently, many deltas are deprived of sediments which in turn leads to coastal erosion and a loss of marshland and mangroves (38, 47, 50); see also (Biggs et al. 2018).

Other variables were also identified by the authors (Appendix SM1.d) as contributor to regime shifts, some pathways leading to social–ecological resilience. For example, change in water temperature variable leads to a change in riverine species composition and involves a consequent shift in farming systems (4, 23), see also Pham et al. (2018). A rise in salinisation leads to a decrease in drinking water sources and has an adverse impact on health and well-being (28, 74, 75). These are major feedbacks that also impact dominant agricultural practices: rice cropping or shrimp aquaculture farming. After crossing the t2 threshold (Fig. 4) the regime shifts with feedback loops, with a major decline in social well-being and ecological functionalities lead to the system's collapse (Fig. 4). The feedback, here the increased production of shrimp, leads to a sudden decline of rice crops which used to be cultivated at the same place. This illustrates how feedback responds to sudden collapses of SESs; here the collapse of rice cultivation and at the same time the decline in freshwater (10, 15, 23, 30, 74, 75, 77), see also Suding et al. (2004) and van de Leemput et al. (2016). The existence of multiple positive feedback loops in the SES inevitably leads to the significant loss of social–ecological resilience with an irreversible collapse of biodiversity, ecosystem goods and services and human well-being (77). Therefore, it is of major importance to anticipate feedbacks, which operate as loops further regulating social–ecological system dynamics and playing a significant role in social–ecological resilience (77); see also (Biggs et al. 2018). Adaptations are still possible (Fig. 4D) with major efforts invested by human societies to regulate their impacts on the earth system and avoid unwanted feedback (85, 60), see also Dearing et al. (2014, 2015).

### Delta tipping point causes, impacts and consequences as exemplified by the top three cited deltas in the reviewed literature

**Fig. 5:**
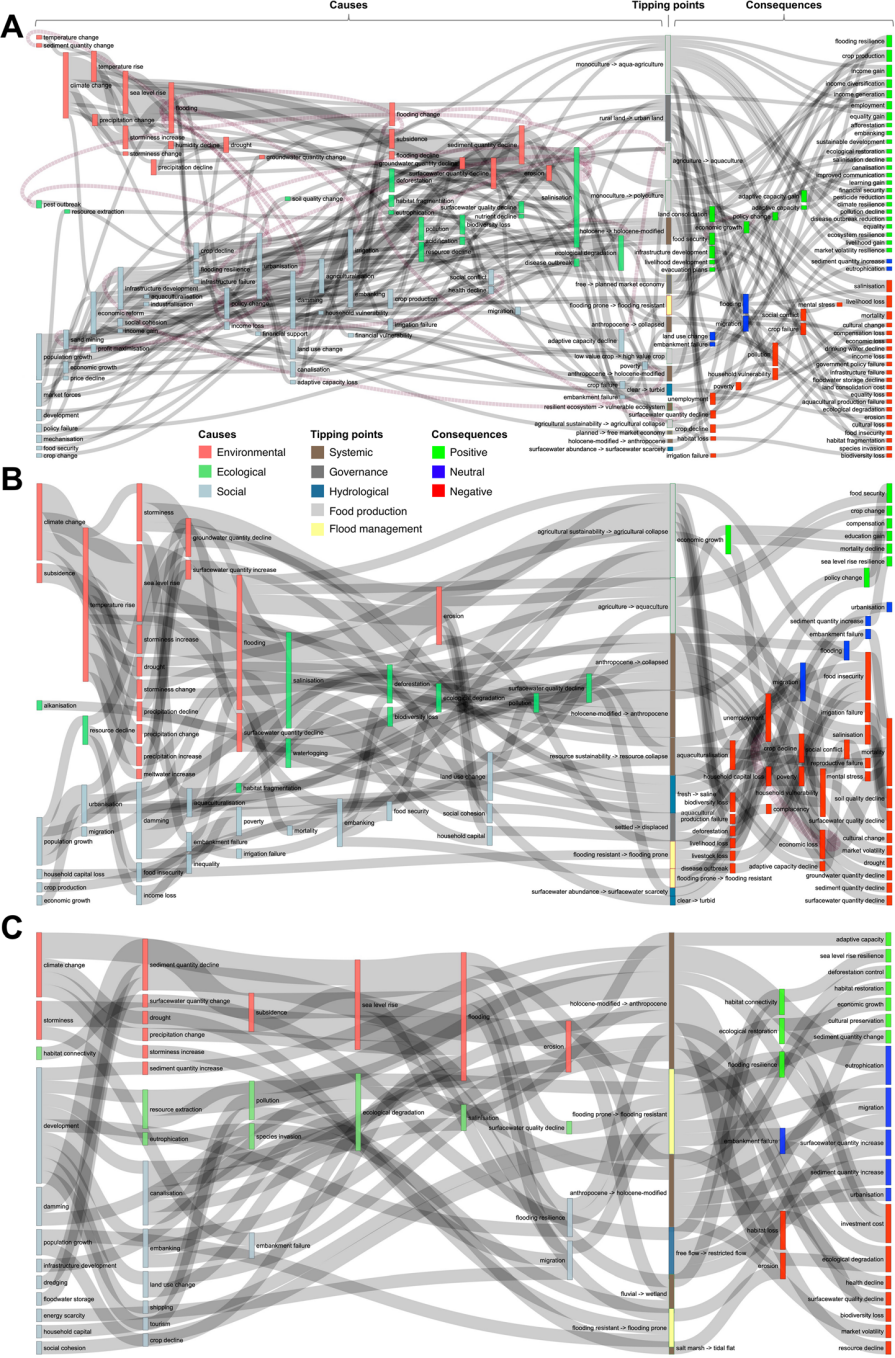
Relationships between the causes and consequences of tipping points for the top three deltas featured in this review: Mekong (**A**), Ganges–Meghna–Brahmaputra (**B**), and Mississippi–Atchafalaya–Wax Lake (**C**) deltas. Only keywords that were mentioned three times or more are shown. Node size is proportional to the number of times the keyword was cited per record. Dashed lines represent feedback loops

The MRD, GBM, and MAW Deltas (see Appendix SM2: Review) were featured most frequently in the review (Fig. 1). For these three mega-deltas, tipping point causes, impacts and consequences were compared (Fig. 5).

Differences in tipping point impacts were apparent between deltas. The consequences of tipping points are mostly negative, with the top three cited being migration, ecological degradation, and habitat loss. Key differences in potential (i.e. predicted) tipping points emerge between the MRD, GBM, and MAW Lake deltas. These potential tipping points are typically related to flood impacts in the MRD (Fig. 5A), damaging systemic changes in the GBM (Fig. 5B, and both systemic (both adaptive and maladaptive) and flood management-related tipping points in the MAW delta (Fig. 5C). Consequences were seen to be predominantly negative in the GBM delta, with health-related factors being commonly cited (e.g. mental stress, poverty, unemployment, and mortality: Fig. 4B), moreover, these tipping points are related to systemic failures in food security (agricultural sustainability → agricultural collapse) and delta-scale state shifts (Anthropocene → collapsed, Holocene-modified → Anthropocene) (Fig. 5B). For the MAW delta (Fig. 5C), key tipping points occurred in both directions (both Holocene-modified → Anthropocene and Anthropocene → Holocene-modified) and flood management (flooding prone → flooding resistant and flooding resistant → flooding prone) (Fig. 5C). These positive and negative tipping points are found in almost equal proportion because of the social efforts within categories of ecological protection (ecological restoration vs. degradation, habitat connectivity vs. fragmentation, afforestation vs. deforestation), environmental change (surface and groundwater quality/quantity gain vs. loss, sediment quantity gain vs. loss), economic prosperity (income gain vs. loss, employment vs unemployment, livelihood gain vs. loss), and social justice (equality gain vs. loss, adaptive capacity vs. mental stress, cultural preservation vs. loss) (Fig. 5C).

## Discussion

Our review found evidence of multiple tipping points being crossed, predominantly at sub-delta/delta and decadal scales. These have driven numerous social, ecological, and economic system transitions in the world’s river deltas. We have evidenced these documented tipping points, their causes, and their consequences on social–ecological systems into categories defined by common properties. Transitions in land use, hydrology, governance, and broader delta-wide systemic shifts were the result of a cascading chain of anthropogenically-induced geophysical and ecological change in the pursuit of social and economic development—as is often observed in other non-delta SESs (Reyers et al. 2018). We discuss below the key features of tipping points in river delta SESs (“River delta tipping point characteristics and entanglement of multiple drivers at different scales” section), how the consequences of tipping points inform the long-term trajectories of deltas (“Current management practices and long-term sustainability of river deltas” section), and which options are available for managing tipping points (“Recommendations” section).

### River delta tipping point characteristics and entanglement of multiple drivers at different scales

As indicated in Section “Results”, river delta tipping points were clustered at singular spatial (sub-delta/delta) and temporal (decadal) scales. This is unique compared to other documented regime shifts (Rocha et al. 2018) where tipping points manifested across scales have been typically harder to manage (Tàbara et al. 2022). Therefore, policy intervention targeted at a range of scales will likely have the largest impact in dictating the nature and direction of delta tipping points (see Appendix SM2: Review, R code). For example, large-scale economic, social, water and land management reform (Zevenbergen et al. 2018; Turley and Selden 2019) have been instrumental in triggering multiple tipping points that had both positive (e.g. economic growth, flooding resilience, and ecological restoration) and negative (e.g. freshwater quality decline, habitat loss, and household vulnerability) consequences for the world’s mega-deltas.

Consideration of multiple and interacting drivers remains often overlooked in the literature (Lenton 2020). This is especially concerning given that the likelihood of tipping points occurring increases when multiple causes are at play (Willcock et al. 2023), and when positive feedback loops exist within the system (Nguyen et al. 2019a, b), potentially leading to accelerating rates and trajectories of change. Interventions at different scales can also concurrently target multiple drivers, increasing the chances of influencing the outcome of multiple tipping points (Rocha et al. 2015; Lauerburg et al. 2020). In river deltas, multiple drivers frequently led to tipping points. For example, 22 unique and often interlinked proximal causes including surface water quality decline, erosion, and habitat fragmentation were responsible for the large-scale shift in deltas from ‘Holocene-modified’ to ‘Anthropocene’ states (See SM2-Nodes).

Whilst multiple tipping point drivers were described here, dominant factors occurring repeatedly including flooding, salinisation, and construction of dams and reservoirs were often responsible for triggering several tipping points throughout delta SESs. By demonstrating how key drivers shape delta tipping points, interventions targeted at these offer pathways to achieving more sustainable delta futures. The drivers of tipping points were also associated with different disciplines of study, spanning cultural studies, hazard management, and ecological literature emphasising the value of a study of this scale involving researchers from the natural and physical sciences, the arts and humanities and the social sciences. A transdisciplinary approach enabled more holistic understanding of the causes, impacts, and consequences of tipping points. Studies have often been discipline-specific, making it difficult to diagnose the causes of tipping points (e.g. Hughes et al. 2013). Our study emphasises that river deltas must be considered as social–ecological systems (Liu et al. 2015) and tipping points are to be properly identified at as early a stage as possible and, thus, managed to the best of our ability.

### Current management practices and long-term sustainability of river deltas

Through a series of tipping points, many river delta SESs appeared to be moving incrementally towards a state of unsustainable development that has led to widespread ecological and social degradation in terms of traditional livelihoods (an ‘Anthropocene’ state). It becomes ever more vital to avoid situations characterised by loss of social cohesion and widespread forced migration (a ‘Collapsed’ state sensu Renaud et al. 2013).

The MAW delta is seen to have experienced tipping points characteristic of a delta transitioning to a modified state, necessitating balancing environmental restoration with flood risk management and socio-economic development on a path towards sustainable development. Historical river management has reduced sediment supply from flooding, preventing the MAW delta from accreting at rates comparable to sea level rise. This subsidence exposes ecosystems and the delta communities that rely on them to greater coastal flooding risk and is a phenomenon observed in many of the world’s deltas such as in the Mekong (35), Nile (21), GBM (9), the Ebro (64), the Rhine–Meuse (8), the Mississippi River deltas (38, 49), see also Twilley et al. (2016). Plans to divert the Mississippi and Atchafalaya rivers and restart sediment deposition within the delta aim to restore a key natural process that would shift the delta to a more sustainable Holocene-modified state over the long term (Xu et al. 2019).

The MRD has undergone major transformations in food production systems with significant impacts on living standards, economic prosperity, traditional food production culture, and environmental health. Whilst several patterns observed were indicative of transitions towards sustainable development (e.g. flooding resilience, income diversification, and reduction in poverty by equality gain), others were indicative of a delta approaching a collapsed state (e.g. salinisation, livelihood loss, and pollution). In April 2022, the Vietnamese government mandated Decision 450—a policy that sets out the nation’s vision for sustainable socio-economic development by 2050 (TVPL, 2023).^1^ The future of the Mekong Delta appears balanced on a knife edge, where the success or failure of policies to reverse widespread food and water insecurity from salinisation (Van Tho 2022), land loss through erosion and sediment diversion (Anthony et al. 2015), and multi-source pollution (Renaud et al. 2013) such as thermal variations of water (Ben-Asher et al. 2016) or microplastic pollution (Kieu-Le et al. 2023) will either tip the delta on a pathway towards sustainability or towards collapse.

The GBM delta displayed many characteristics of a collapsed state, primarily associated with social decline (e.g. unemployment, household vulnerability, food insecurity, and mortality) and environmental degradation (principally biodiversity loss, soil quality decline, and salinisation) linked to agricultural collapse (Szabo et al. 2016b; Nicholls et al. 2020). Solutions such as Tidal River Management (Adnan et al. 2020) and mangrove restoration (Banerjee et al. 2023) seek to reinstate natural water–sediment processes, mitigating flood and erosion hazards that are the root causes of the many food production tipping points described in this review.

Efforts to move deltas towards sustainable states are laudable; however, the challenge of doing so may be insurmountable. Increasing costs of energy and material under growing climate instability make sustainable transitions of already degraded delta systems ‘very challenging’ for the MAW, MRD, and GBM deltas (Day et al. 2016). Most river delta SESs may even be locked into their unsustainable state, heading for collapse unless transformative action can be instigated (Wesselink et al. 2020). Deltas in wealthy nations are expected to fare better, given the economic capacity to maintain existing infrastructure and finance transitions towards more sustainable futures. Even then, increasing climate vulnerability, especially in already flood-prone regions such as the MAW delta, makes this increasingly unlikely (Tessler et al. 2015; Haasnoot et al. 2019). The landward retreat may, ultimately, be the fate of many delta communities that cannot adjust to future sea levels (Ibáñez et al. 2014; Magnan et al. 2022).

The social–ecological system of each of the three river deltas discussed above can conceptually be placed along a continuum between a “utopian” future, where social and ecological processes interact harmoniously, and a “dystopian” future, where environmental collapse due to prolonged unsustainable development makes deltas essentially uninhabitable (Fig. 5). If all the world’s river deltas were placed along such a scale (e.g. Renaud et al. 2013), all would likely fall far from a utopian state. Increasing energy and material costs under growing climate instability make sustainable transitions of already degraded delta systems very challenging for the MAW, MRD, and GBM deltas (Day et al. 2016).

Disregarding political factors can lead to a failure to initiate and implement reforms and larger-scale transformations (Seijger et al. 2019; Wesselink et al. 2020) and dual lock-in of technological and institutional systems act as constraints for moving into a more sustainable direction over the longer term (Wesselink et al. 2020). Co-management and integrated river management require consensus amongst political views and power positions of varied actors, which is hard to achieve (Tran et al. 2019; Phong et al. 2023).

## Recommendations

Recommendations to aid management decisions related to tipping points identified in this research are presented in Table 1.

**Table 1:** Recommendations proposed in the reviewed papers on how to reduce the effects of negative tipping points and promote positive tipping points

	Recommendations	References by ID number
Reduce negative tipping points	**Monitoring**: understand the delta’s unique socio-ecological landscape through **better monitoring**, and modelling of socio-ecological system dynamics—future modelling should consider identifying extremes and/or system-changing thresholds in natural and human environments, with approaches such as horizon scanning. This involves improving the monitoring network to further understand the influence of surface and groundwater on the ecology, understanding vegetation cover (including salt marshes edge), patchiness dynamics through its hydrogeomorphic and biogeochemical implications and ecosystem’s interactions with intensifying human-induced stressors.	32: Baigún et al. (2008), 68: Chen et al. (2021), 56: Bush et al. (2010), 42: Cui et al. (2010), 84: Lomeli-Banda et al. (2021), 55: Silliman et al. (2016), and 62: Wright et al. (2018)
	**Releasing freshwater and sediment from upstream: the upstream freshwater supply is necessary for the protection of the mangrove wetland ecosystems in the river deltas.** This may involve large scale infrastructure removal or fundamental alteration of basic infrastructures such as dikes and dams or engineering rethinking of infrastructures—which requires investments. Restoring rivers involves the overall understanding of the coastal environment's multiple state dynamics. **Regulating and controlling groundwater** extraction is needed to avoid subsidence, groundwater salinisation and the accumulation of pollution.	24: Garschagen et al. (2011), 4 & 23: Hossain et al. (2017, 2020), 50: Jensen and Morita (2020) 74/75: Kumar et al. (2020), 8-12: Renaud et al. (2013), 13: Hoan et al. (2019), and 52: Sherin et al. (2020)
	**Regulating land use to avoid smallholders’ land loss and enhancing the protection and conservation of ecosystems through policies** is needed to reduce industrial forcing on landscapes. Developing effective measures of habitat protection must be planned in regard of communities’ livelihoods. Appropriate identification and prioritisation of areas designated for conservation may provide means of reaching the long-term conservation goal only if it considers the social well-being. This includes respecting the existence of a connectivity threshold as well as communities’ livelihood to maintain social–ecological systems.	24: Garschagen et al. (2011), and 84: Lomeli-Banda et al. (2021)
	**Learning and knowledge exchange between stakeholders (social learning):** improving social learning processes through the concertation of all stakeholders (improved governance). Communities’ knowledge should be considered in future planning and design of urban and rural areas. It is important to cultivate bi-directional communication between researchers and stakeholders to help improve the relevance and applicability of the findings. This involves understanding actors’/stakeholders’/residents’ perceptions of environmental changes and integrating their ecological knowledge and adaptation measures, into development planning.	87: Nhat Lam Duyen et al. (2021), 4: Hossain et al. (2020), 38: Lam et al. (2018), 51: Long et al. (2020), 29: Shinn (2018), 58: De Lima et al. (2020), 70: Allen (2021), and 71: Lawson et al. (2021)
	**Empowering to adapt** through **participatory co-management approaches:** community engagement and participatory engagement and dialogues among scientists and stakeholders can help to improve water resource governance. **Empower smallholders** with different livelihood options and promote agronomic diversification.	29 & 54: Shinn et al. (2014); Shinn (2018), 77: Kattel (2020), 38: Lam et al. (2018), and 7: Paille et al. (2016)
	**Scale**: understanding the interactions between small-scale and large-scale tipping points—understanding hydrological processes are critical to define the functions and values of wetlands, vegetation community evolution since its creation, and the ecological processes happening.	2: de Araujo Barbosa et al. (2016), 24: Garschagen et al. (2011), 27: Deb and Haque (2011), 66: Nijhuis et al. (2021), 70: Allens (2020) and 43: Bargu et al. (2019)
Enhance positive tipping points	**Integrated approaches for river and coastal zone management**. The integrated approach involves the use of indices to measure the riverine ecosystem health by resource managers as a part of the Integrated Water Resources Management program. Involving interdisciplinary approaches in the management plan can support the long-term conservation and protection of mangrove wetland ecosystems (Islam and Gnauck 2008). This includes the adoption of socio-hydrological concept that gives an equal weight to science (hydrology, meteorology, etc.) and social components (citizen participation, law) to co-design robust solutions for adaption or mitigation measures.	47: Islam and Gnauck (2008), 28: Liu et al. (2018), 51: Long et al. (2020), 43: Bargu et al. (2019), 68: Chen et al. (2021), 50: Jensen and Morita (2019), 77: Kattel (2020), 74&75: Kumar et al. (2020), and 28: Liu et al. (2018)
	**Restore coastal ecosystems and implement green infrastructures** (including mangrove and salt marshes restoration). Vegetated wetlands within islands are typically hotspots of land growth and biological nutrient removal, which can counteract land loss and prevent the expansion of coastal oxygen-depleted zones.	26: Day et al. (2019), 68: Chen et al. (2021), 14: Le et al. (2020), 47: Islam and Gnauck (2008), 42: Cui et al. (2010) and 62: Wright et al. (2018)
	**Develop climate-smart infrastructures to allow rivers to bring new sediments:** e.g. rethink polders and other Tidal River Management.	34: Minar et al. (2013), 26: Day et al. (2019), 53: Allison et al. (2017), 31: Amer et al. (2017), and 72: Rahman et al. (2021)
	**Climate-smart agriculture**: climate resilient aquaculture practices, landscape integrated farming (e.g. Silvo-fisheries), integrated production practices/rice-fish, rotation of crops (rice, shrimp, corn) with mung bean crops, mangrove-shrimp.	19 & 20: Marchand et al. (2008), 45: Deinne and Ajayi (2021), 56: Bush et al. (2010), 78: Dou et al. (2020), 46: Dubey et al. (2017), and 82: Freed et al. (2020)
	**Improve the land-use planning**, actions, and implementation of well justified river management policies; Expert elicitation with an in-depth polling of experts on issues with high uncertainty or controversy; dynamic planning needs to be associated with monitoring. **Invest in livelihood development through government**, equality and poverty reduction, public services, access to basic amenities to reduce social inequality (and all SDGs). **Negotiate multilaterally with the transboundary nations** to prevent the ecological collapse of the fish stock is urgently needed. A carefully designed international cooperative governance framework is also thought to protect water resources and sustain ecosystem goods and services.	56: Bush et al. (2010), 73: Norgaard et al. (2021) 45: Deinne and Ajayi (2021), 78: Dou et al. (2020), and 83: Pham et al. (2017), and 77: Kattel (2020)

To understand and model complex delta SESs, the scientific monitoring of hydrological and ecosystem states, and research on the social dimensions of tipping points, would benefit from financial and technical support from international, national and local institutions (32, 42, 55, 56, 62, 68–69, 84), see also Brondizio et al. (2016). Among other parameters leading to tipping points, additional research on saline water intrusion and soil salinity (18, 39, 48, 52, 67, 74–75), see also Abdullah et al. (2019), Akter et al. (2019),  Das et al. (2020), arsenic and heavy metals (36), groundwater depletion (8–12, 13, 74–75, 52) see also Minderhoud et al. (2017) is required. Additional investments in monitoring subsidence by measuring total surface elevation change, e.g. by Interferometric Synthetic Aperture Radar (InSAR), Light Detection and Ranging (LiDAR) or Global Positioning System (GPS), and in situ, subsidence is essential to facilitate management decisions in subsiding deltas (Minderhoud et al. 2017, 2018, 2020).

Based on the result of research and monitoring, a consensus appears in the literature reviewed for greater releases of both fresh water and sediment from dams (4, 23, 24, 50). This recommendation is also supported by a broader research community which was not included in the review (e.g. Syvitski 2008; Syvitski et al. 2009; Tessler et al. 2015, 2018). The fresh water and sediment release is required to be done through concerted basin scale and—where applicable—transboundary efforts between nation states to ensure benefits for communities located downstream. At the same time, effective delta-level policy strategies can support better control and regulation of the extraction of groundwater. This is imperative to limit the extraction for agricultural and industrial purposes leading  to delta subsidence, and avoid the salinisation and the pollution of groundwater (8–12, 13, 74–75, 52), see also Minderhoud et al. (2017, 2018, 2020).

Structural changes in natural and social components of deltas, emerging from past land-use changes, have led deltas to become ‘locked-in’, thus, losing the ability to transform back into ‘living’ (i.e. dynamic) deltas’ and consequently, making them more at risk to shocks (Santos and Dekker 2020). Regulating land use and enhancing the protection and conservation of ecosystems through policies should be done whilst giving full respect and priority to local communities whose livelihoods depend on natural resources and ecosystem services provided by delta forests and wetland systems (5, 25, 32, 36, 50, 57, 62, 68). To avoid negative tipping points and enhance social–ecological resilience, i.e. the ability of a system to persist in the face of perturbations, the literature suggests considering the social context and ecological context together and on the same basis (4, 29, 38, 58, 59, 70, 71, 88–87).

Furthermore, tipping points can be avoided through efficient learning and knowledge exchange between stakeholders (4, 29, 38, 58, 59, 70, 71, 88–87). Improving social learning processes would benefit from better cooperation, participation, and engagement of all stakeholders (improved governance). Farmers and citizens could be involved in the monitoring with the support of scientists and co-produce sustainable solutions. In Vietnam’s Mekong River Delta, some farmers are monitoring the salinity levels in their fields. They respond to saline-induced reductions in rice productivity by practising integrated farming that benefits communities and natural resources management (Leigh et al. 2020; Thanh et al. 2020; Van Kien et al. 2020). Such progress in the field of sustainable management of SESs requires rethinking of the values of the relations between Nature and Society and implementing governance arrangements for better participation and engagement of all stakeholders (Pascual et al. 2023). Through the engagement of all stakeholders, a balance between strengthening ecosystems and safeguarding their services or contributions to communities can be found.

In addition, the implementation of locally led plans can highly benefit from the empowerment of smallholders within local communities to monitor the ecological conditions of the ecosystems (Ranjan 2019). This is possible through the active engagement of all actors within local communities enhanced by participatory co-management practices (7, 29, 38, 54, 77). Community-level information and citizen observation collected with approaches such as citizen science (examples from Moorhouse et al. 2021) or other participatory methods can be meaningfully incorporated into each delta state (Reyes-García et al. 2020; Syvitski et al. 2022). Capacity-building can enhance smallholders socio-economic resilience. Ultimately, the engagement of all actors in the definition of the regulation of land use could avoid or limit human impact on the landscapes without dispossessing anyone of the resources needed to sustain livelihoods (Dewan and Nustad 2023). Therefore, the relations of power within local communities, involving inequalities, discriminations and dispossession need to be identified in the phases of concentration for the construction of future landscapes to leave no one behind (de Micheaux et al. 2018; Kumar et al. 2020; Mukherjee and Ghosh 2020). Conservation and restoration cannot be achieved without consideration of social justice (Washington et al. 2018).

To enhance positive tipping points, the literature typically recommends enacting policies that integrate river and coastal zone management (e.g. 47), restoring and deploying green infrastructures (e.g. 26), supporting climate-smart agriculture (20, 45, 46, 56, 78, 82) and agro-ecological practices, improving land-use planning, investing in diversified livelihood development, and improving transboundary negotiations for river management. Integrated river management recognises and includes local ecological knowledge that supports sustainable practices (Shah and van Koppen 2016;  Sebesvari et al. 2017; den Haan et al. 2019; Benson et al. 2020; Ladel et al. 2020; Franzke et al. 2022). Supporting knowledge exchange and learning within communities and between locally rooted stakeholders is now highly recommended in the process leading to deltas’ “locally led adaptation” (Rahman et al. 2023a, 2023b).

The literature is overall critical of engineering solutions (such as raising embankments or sea walls) proposed as a sole option to increase SES resilience (50, 58, 59, 70), see also Sarkar et al. (2016), Gain et al. (2019), Ghosh and Mistri (2020), Chaudhuri et al. (2022). To restore coastal ecosystems and implement green infrastructures (including mangrove and salt marsh restoration) and find solutions based on ecosystem or nature-based solutions, the literature recommends improving the monitoring of coastal delta geophysical dynamics from the river basin scale to more local scales (Kumar et al. 2021; Banerjee et al. 2023). Working with people and nature can help in developing sustainable solutions, especially for sediment management(Darby et al. 2015, 2020; Dunn et al. 2019; Best and Darby 2020; Gain et al. 2022). Applied approaches need to be holistically assessed to maximise benefits at the local scale. For example, in Bangladesh, some embankment infrastructures can slow down coastal erosion processes and could help reconstruct marshland, but at the same time, experiments with Tidal River Management (TRM: a form of poldering) appear to be controversial as it results in the temporary loss of productive land and hence livelihoods (Gain et al. 2019). Without addressing the short-term institutional limitations and providing compensation, many communities are reluctant to implement TRM (Gain et al. 2019). The competing interests between hazard exposure, economic development, social welfare, and environmental protection make delta planning more complex, and a clear understanding of trends, threats and trade-offs is essential (Suckall et al. 2018).

Finally, defining alternative sustainable practices to avoid negative tipping points is difficult considering spatial and temporal aspects of delta transformation, especially in large and complex delta systems. Interactions between subsystems and system components in delta SESs are highly complex and dynamic and are, thus, difficult to characterise in management terms. Timescales are important to consider as rapid and short-term economic activity may have a long-term impact on ecosystems. Action can be taken in the short term to avoid a particular tipping point, but long-term time frames are frequently needed to see the benefits of ecosystem restoration. To avoid negative tipping points and to ensure the sustainability of social–ecological systems, it is important to design management and adaptive pathways that balance ecological, social, economic and governance aspects equally. Recognising early warning signals that indicate when thresholds are being approached is still a major challenge (Scheffer et al. 2009; Dakos et al. 2013). Identifying change drivers as an early warning signal would help in anticipating  and allow more time to potentially intervene in the processes leading to negative tipping points.

These recommendations would require intense efforts in the orientations of policies and deciding on trade-offs between economic and ecological dynamics. For example, releasing freshwater or removing dams will reduce water for irrigation during the dry season and hydroelectricity production but will support transport of sediments and water from upstream to downstream (Garschagen et al. 2011; Hossain et al. 2017, 2020; Jensen and Morita 2020). Some recommendations suggest regulating land use and enhancing the protection and conservation of ecosystems through management and policies while others suggest ecosystem restoration, and support for ‘smart’ agriculture (Yáñez-Arancibia and Day 2004; Chong 2006; Baigún et al. 2008; Darby et al. 2016; Karczmarski et al. 2017; Wright et al. 2018; Bai et al. 2019; Jensen and Morita 2020; Chen et al. 2021). Most of these policies aiming at reducing negative tipping points or enhancing positive ones would be more successful (a) if coupled with an improved social learning process where stakeholders can share and learn from each other's experience of sustainable practices, and (b) if  all stakeholders are engaged with and participate in the design and the implementation of policies.

## Conclusion 

Many deltas worldwide have now been or are being transformed from a modified-Holocene state towards a fully Anthropocene state. These transformations, when exacerbated by the effects of climate change, could push some deltas past tipping points toward an unfavourable future. Our cross-system analysis based on an in-depth literature review process has shown that tipping points can occur across different scales of both time and space and within regional or local social–ecological systems. We have identified a wide range of drivers within interlinked social–ecological systems. Recommendations centre around how sustainable agriculture, ecosystem services and nature-based solutions should be considered in the planning of delta infrastructure and integrated into engineering solutions to benefit Anthropocene delta SESs (Brondizio et al. 2016). Multi-stakeholder collaborations from the local to the global level already support the development of advanced governance mechanisms. Capacity-building is also an important tool in identifying and understanding tipping points, especially in large deltas in the context of developing economies (van Nieuwaal et al. 2023). In addition to a fair and adaptive governance system, co-produced scientific collaborations between academic and non-academic stakeholders can help to generate information on the past, present, and potential future social–ecological conditions of the deltas, as a part of an upstream–downstream management system. Additional research on the development of tools, improving the modelling of hazards, sharing data to strengthen the assessment of the vulnerability of social–ecological systems, and providing access to information for stakeholders is urgently required.

Identifying feedback and non-linear responses remains key for developing sustainable solutions to improve delta ecological health and human well-being. Implementing adaptive management on its own might not be sufficient if delta social–ecological tipping points are reached; considering recent indications that the global future of deltas is ‘perilous’ (Haq and Milliman 2023) efforts must be made in all aspects to avoid negative and irreversible delta tipping points and the loss of sustainable livelihoods in the world’s most vulnerable landforms.

### Supplementary Information

Below is the link to the electronic supplementary material.

Supplementary file1 (PDF 747 kb)

Supplementary file2 (XLSX 1056 kb)

## Footnotes

1 TVPL (2023) Accessed 27/06/23. https://thuvienphapluat.vn/van-ban/Tai-nguyen-Moi-truong/Decision-450-QD-TTg-2022-Approving-national-environmental-protection-strategy-until-2030-510740.aspx.
