# Supplementary material for: Causes and consequences of tipping points in river delta social–ecological systems
Source: Ambio. 2024 Apr 13;53(7):1015–36. doi: 10.1007/s13280-023-01978-2 (PMC11101396; doi:10.1007/s13280-023-01978-2)
Supplement: Supplementary file 1 — Supplementary file1 (PDF 747 kb) [file 13280_2023_1978_MOESM1_ESM.pdf]

Ambio

Electronic Supplementary Material
This supplementary material has not been peer-reviewed.

Title: Causes and Consequences of Tipping Points in River Delta Social-ecological Systems

Authors: Emilie Cremin, Cai J. T. Ladd, Thorsten Balke, Sumana Banerjee, Ly H. Bui, Tuhin Ghosh, Andy Large, Hue Thi Van Le, Kien V. Nguyen, Lan X. Nguyen, Tanh T. N. Nguyen, Vinh Nguyen, Indrajit Pal, Sylvia Szabo, Ha Tran, Zita Sebesvari, Shah Alam Khan, Fabrice G. Renaud

SM1. Literature review's process and summary

Table of Contents

SM1. Literature review's process and summary **1**

SM1.a. Keywords and definitions per qualifier group used to identify peer-reviewed literature on socio-ecological tipping points in river deltas, published up until 19/10/21 and recorded in the Web of Science and SCOPUS databases. Papers were selected based on the title, keywords, and abstract. **2**

SM1.b. Process of the literature review **3**

SM1.c. Number of studies featured in the review **4**

SM1.d. Biophysical thresholds for ecosystems and agro-ecological systems **5**

The table presents the main biophysical thresholds identified in the papers. Exceeding these thresholds suggests that SES may lose resilience soon and increase the likelihood of regime shifts (Hossain et al., 2020). **5**

SM1.a. Keywords and definitions per qualifier group used to identify peer-reviewed literature on socio-ecological tipping points in river deltas, published up until 19/10/21 and recorded in the Web of Science and SCOPUS databases. Papers were selected based on the title, keywords, and abstract.

Qualifier	Keyword	Definition
**Geography**	delta*	A geographic unit. Deltas are a landform created by deposition of sediment that is carried by a river as the flow leaves its mouth and enters an ocean, sea, estuary (Syvitski, 2008). Therefore, this key word refers to a geographical region, characterised by a river system and connected to the ocean (region, river, ocean) (Dunn et al., 2019).
**System**	ecologic* OR soci*	The concept of a social-ecological system recognizes the feedback that occurs between social and ecological sub-systems. We define a social-ecological system as complex, integrated systems in which humans are part of nature an ecological system or biological units interacting with a social system (adapted from Berkes et al., 2003; Berkes and Folke, 1998, 2000; Folke et al., 2016a, 2016b; Gain et al., 2021; Gunderson et al., 2017).
**Theory**	(“adaptive$capacit*” OR bifurcat* OR “catastrophic$shift*” OR “critical$transition*” OR flicker* OR hyster* OR panarch* OR “regime$shift*” OR resilien* OR “*stable$state*” OR threshold* OR “tipping$point*”	Concepts of adaptive capacity (Dixit and Moench, 2004), adaptive cycle, alternative stable state (Suding et al., 2004), bifurcation point (Ashwin et al., 2012; Thompson and Sieber, 2011), cascade effect, panarchy (Gunderson and Holling, 2001; Ostrom, 2004), paradigm shift, regime shift, Threshold change, Tipping point (Carpenter et al., 2011; Folke et al., 2004; Gunderson et al., 2017; Lenton, 2011, 2020; Lenton et al., 2008, 2012, n.d.; Scheffer and Carpenter, 2003; Walker et al., 2004) were identified as key words. These refers to the definitions of Qualifier (Adaptation, Change, Collapse, Disturbance, Failure, Irreversible, Perturbation, Regeneration, Rehabilitation, Resilience, Sustainability, Transition, etc.). The qualifier had to be related to transformative processes as mentioned by the IPCC, 2021 report (Masson-Delmotte et al., 2021).

SM1.b. Process of the literature review

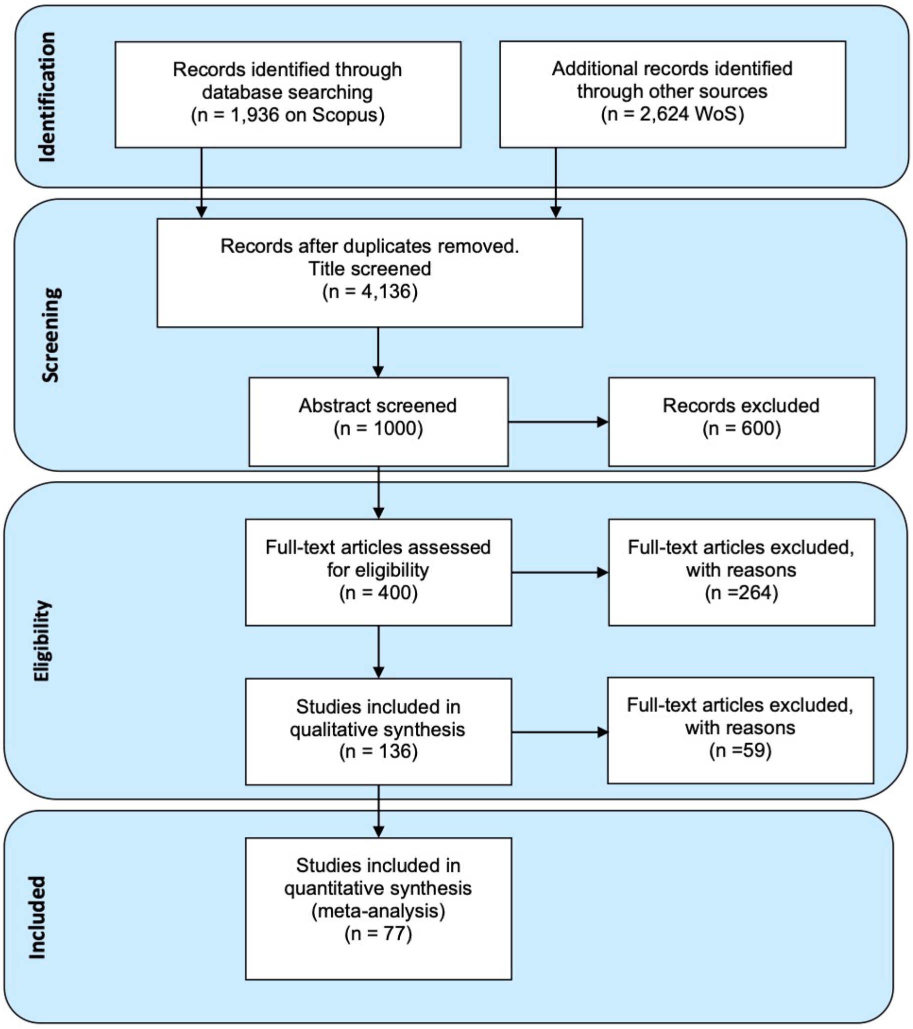

Template from: Moher D, Liberati A, Tetzlaff J, Altman DG, The PRISMA Group (2009). *P*referred *R*eporting *I*tems for *S*ystematic Reviews and *M*eta-*A*nalyses: The PRISMA Statement. PLoS Med 6(7): e1000097. doi:10.1371/journal.pmed1000097

For more information, visit www.prisma-statement.org.

SM1.c. Number of studies featured in the review

Ambio

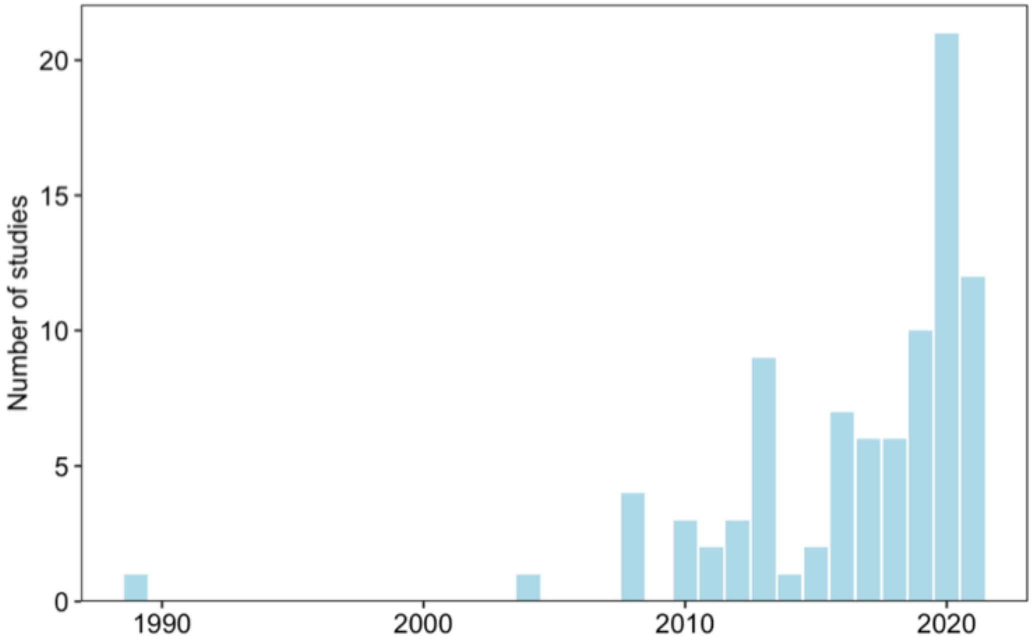

Number of studies per year featured in this review. Spatio-temporal scale of tipping point studies

SM1.d. Biophysical thresholds for ecosystems and agro-ecological systems

The table presents the main biophysical thresholds identified in the papers. Exceeding these thresholds suggests that SES may lose resilience soon and increase the likelihood of regime shifts (Hossain et al., 2020).

**Elements of the social-ecological systems (Biomes)**	**Types of ecosystems and agro-ecological systems**	**Threshold**	**Tipping point and regime shift**	**Adaptation**	**References**
A – River ecosystems	River Delta, lakes, creeks, lagoons (lakes with brackish water),	Retention of water flow and sediment discharge by infrastructures.<br><br>Sand mining in the riverbed.<br><br>Drainage of wetlands, leaving the delta susceptible to sea level rise<br><br>Geomorphological erosion<br><br>Alteration of the freshwater supply/quality of water	Alteration of water inflow = disconnections and loss of hydrological connectivity and functionality<br><br>Alteration of sediment flow and discharge = Riverbank and coastal erosion = land loss<br><br>The water and sediment inflow necessary to sustain the hydrology needs for ecosystem's survival depend on the surface of the delta.	Removal of infrastructures to let the water inflow and sediment depositions.<br><br>Maintaining a minimum influx of water – depending on the season, Evapotranspiration rates and sea water level rising<br><br>Transformation of local production systems (diversification, abandonment).<br><br>Adaptations possible but still a decrease of human well-being.	(Islam and Gnauck, 2008; Jensen and Morita, 2020; Kattel, 2020; Lam et al., 2018; Le et al., 2020; Leauthaud et al., 2013; Lomeli-Banda et al., 2021; Marriner et al., 2012; Prado et al., 2019; Zhang et al., 2020)
	Lakes, ponds and in channels during the dry season	eutrophication caused by nutrient enrichments.	Decline of surface water quality	Reduce the quantity of fertilizers	(Kattel, 2020) (Day et al., 2008).
B - Coastal ecosystems	Lagoons	Increasing evapotranspiration and drought due to temperature rising condition	Lack of surface outflow cause a water surface disconnectivity between wetland and sea this involves a reduction in marshlands		(Prado et al., 2019)
	Marshes	The sea-level rising on salt marshes wetland could change the hydrodynamic relationship	In the Colorado River Basin and delta, the upper salinity limits that marsh vegetation could		(Liu et al., 2018; Lomeli-Banda et al., 2021; Pham et al., 2018; Wright et al., 2018)

		between the wetland and the sea. Sea level in the PRD has increased by about 1.4 mm/yr over the past decade (Liu et al., 2018) Below a 'disconnectivity threshold' of 40-50% preferential flow paths develop on vegetated islands which alter hydraulics and residence time	tolerate (6 g L−1) (Baeza et al., 2013).		
	Marshes, marsh, mudflats, and other types of wetlands	The deposition of sediment involves significant habitat gain and regenerative processes / in contrary, the lack of sediment deposits involves soil reduction, habitat losses and fragmentations.	Sediment deposits needs to be higher than the rate of erosion		(Biggs et al., 2018; Suding et al., 2004),
	Marshes, marsh, mudflats, and other types of wetlands	Oil spill on marsh involves the degradation of vegetation where plant's stems are covered by oil, the erosion increases for ~1–2 years after the spill.	None as such, but approximately 18 months after the DWH spill, the increased erosion rates in the highest stem oiling level were no longer detected.		(Silliman et al., 2016)
	Mangrove Forest	~40,000 dS m−1 (Water salinity) 2000 m3 s−1 (Water flow) 25-28 °C, 35 °C (Air temp) 27 °C to 29 °C (Water temp) 0–5 ppt (Soil salinity) ~1500 m3 s−1 (Water flow)	Reduction of forest cover and fragmentation / Deforestation	Conservation with co-management	(Hossain et al., 2020; Islam and Gnauck, 2008)
A and B – Ecotones between coastal and river biomes	Habitats for Wildlife: fauna and flora biodiversity	Habitat degradation and fragmentation due to alteration of near-shore environments / habitat destruction through urban and or industrial coastal developments, modification of	Decline in the freshwater resources including the species diversity / When the effective carrying capacity of each habitat fragment	Protection and Conservation of habitat (over sufficiently large areas), regulation of sea traffic and fishing for habitat quality – secure travelling corridors and afford functional	(Karczmarski et al., 2017; Kattel, 2020)

		shorelines and land reclamation<br>coastal overexploitation (Over-fishing)<br>Ocean acidification	becomes lower than MVPk (minimum viable population in carrying capacity, MVPk)	connectivity to increase their effectiveness	
C- Agricultural land, aquaculture, and fish capture	Fruits orchards	28 C° (Air temp)<br>4 dS m-1 (soil salinity)<br>(~4 to ~10 dS m−1)	Reduction in crop production (quality and quantity)		(Hoan et al., 2019)
	Rice	27/28 C° (Air temp)<br>2 dS m-1 (soil salinity)<br>Decline in soil fertility despite the use of fertilisers	Reduction in crop yield: rice yield will decline by ~18 and ~25%, respectively, for rising temperatures of 2 and 4 °C<br>each unit (ds/mL) rise of salinity causes roughly 12% reduction in rice yield.	Some modern rice varieties could resist soil salinity up to 4 dS m−1 (Hossain et al. 2020)<br><br>Shifting farming system, interaction between the Governmental and farmers (Pham et al. 2017)	(Hossain et al., 2020; Kumar et al., 2020; Pham et al., 2018)
	Shrimp	25–32 °C (Water temp)<br>7.80–39 dS m−1 (Soil salinity)	Reduction in shrimp harvest: above these threshold ranges, shrimp production declines by at least 50% because of virus outbreaks in shrimp farms.		(Hossain et al., 2020)
	Fisheries	27 °C to 29 °C (Water temp)<br>0–5 ppt (Soil salinity)<br>~1500 m3 s−1 (Water flow)<br>A salinity level of 12 dS m−1 to 15 dS m−1 is the limit for fish production in ponds.	Reduction in fish harvest: Fish production reduces by at least 50% at a temperature of 32°C and 15% reduction when 29 °C is exceeded.<br>Algal blooms in ponds (chattenella marina)		(Hossain et al., 2020)
D- Urban systems and settlements	Water consumption	Underground water salinization; water pollution; saline water intrusion affects the security of regional water supply	Salinity exceeded the acceptable threshold for drinking purposes of 250 mg/L.; according		(Kumar et al., 2020b; Liu et al., 2018)

			to the National Hygienic Standard for Drinking Water (GB 5749-2006) (Liu et al. 2018). Reduced access to water (for woman): water prone disease (stroke and cardio-vascular disease) and increased social vulnerability (poverty, mental stress, migrations)		
